# When Trauma Unmasks a Congenital Anomaly: Incidental Discovery of Bilateral Pulmonary Sequestration

**DOI:** 10.7759/cureus.114800

**Published:** 2026-08-19

**Authors:** Houda Bennani, Yahya Elharras, Ouiam Taibi, Ittimade Nassar, Kaoutar Imrani

**Affiliations:** 1 Diagnostic Radiology, Ibn Sina University Hospital, Rabat, MAR; 2 Neuroradiology, Ibn Sina University Hospital, Rabat, MAR; 3 Radiology, Ibn Sina University Hospital, Rabat, MAR

**Keywords:** case report, congenital lung malformation, ct angiography, incidental finding, pulmonary sequestration

## Abstract

Pulmonary sequestration is a rare congenital bronchopulmonary malformation in which dysplastic, non-functioning lung tissue has no or a normal connection with the tracheobronchial tree and is supplied by an aberrant systemic artery. Bilateral involvement is exceptional, and its incidental discovery during trauma imaging is rarely reported; this case, therefore, adds to the limited literature on bilateral sequestration and illustrates how an unrelated indication can unmask a long-silent congenital anomaly. A 35-year-old man with a history of unexplained chronic cough was admitted after a road traffic accident causing closed chest trauma; examination was unremarkable apart from anterior chest pain. Non-enhancing thoracic computed tomography (CT) showed a displaced sternal fracture, together with bilateral cystic and consolidative masses of the lower lobes. A contrast-enhanced CT angiography then demonstrated aberrant systemic arteries arising from the descending thoracic aorta and feeding both lesions, establishing the diagnosis of bilateral intralobar pulmonary sequestration. The sternal fracture was managed conservatively; surgical resection of the sequestration was proposed but declined by the patient, who was placed under regular clinical and radiological surveillance. The take-away lessons are that pulmonary sequestration should be considered before any atypical, bilateral basal lung lesion, that CT angiography is the key examination because it directly depicts the aberrant systemic supply, and that recognizing this entity avoids misdiagnosis as a tumor and unnecessary invasive procedures.

## Introduction

Pulmonary sequestration is a rare congenital anomaly of bronchopulmonary development, accounting for approximately 0.15-6.4% of all congenital pulmonary malformations [1,2]. It consists of dysplastic, non-functioning lung tissue that has no or an aberrant connection with the normal bronchial tree and is supplied by an anomalous systemic artery, most often arising from the thoracic or abdominal aorta [3]. Two anatomical forms are classically distinguished: intralobar and extralobar sequestration (ELS) [2]. Although the malformation is congenital, it is frequently detected only in adolescence or adulthood, when it may present with recurrent pneumonia, chronic cough, or hemoptysis. Because these features are non-specific and may mimic neoplastic or chronic infectious lung disease, sequestration is a recognized source of misdiagnosis and diagnostic delay [2,4].

Multidetector computed tomography (CT), particularly contrast-enhanced CT angiography, is now the imaging modality of choice, as it allows detailed assessment of both the pulmonary parenchyma and the aberrant vascular supply whose demonstration is the key to a confident diagnosis [5]. Bilateral sequestration is exceptional, with only isolated cases reported [6,7]. We report an unusual case of bilateral intralobar pulmonary sequestration incidentally revealed by contrast-enhanced CT angiography performed in a trauma setting.

## Case presentation

A 35-year-old man was admitted to the emergency department after a road traffic accident causing closed chest trauma. His only relevant medical history was a long-standing cough that had never been investigated. He reported no fever, hemoptysis, recurrent respiratory infection, or prior thoracic surgery, and there was no notable family, genetic, or psychosocial history. His primary concern on presentation was post-traumatic anterior chest pain.

On admission, the general condition was preserved, and the patient was hemodynamically stable. Physical examination was unremarkable apart from tenderness of the anterior chest wall; pulmonary auscultation was normal, and there were no signs of respiratory distress.

**Figure 1:**
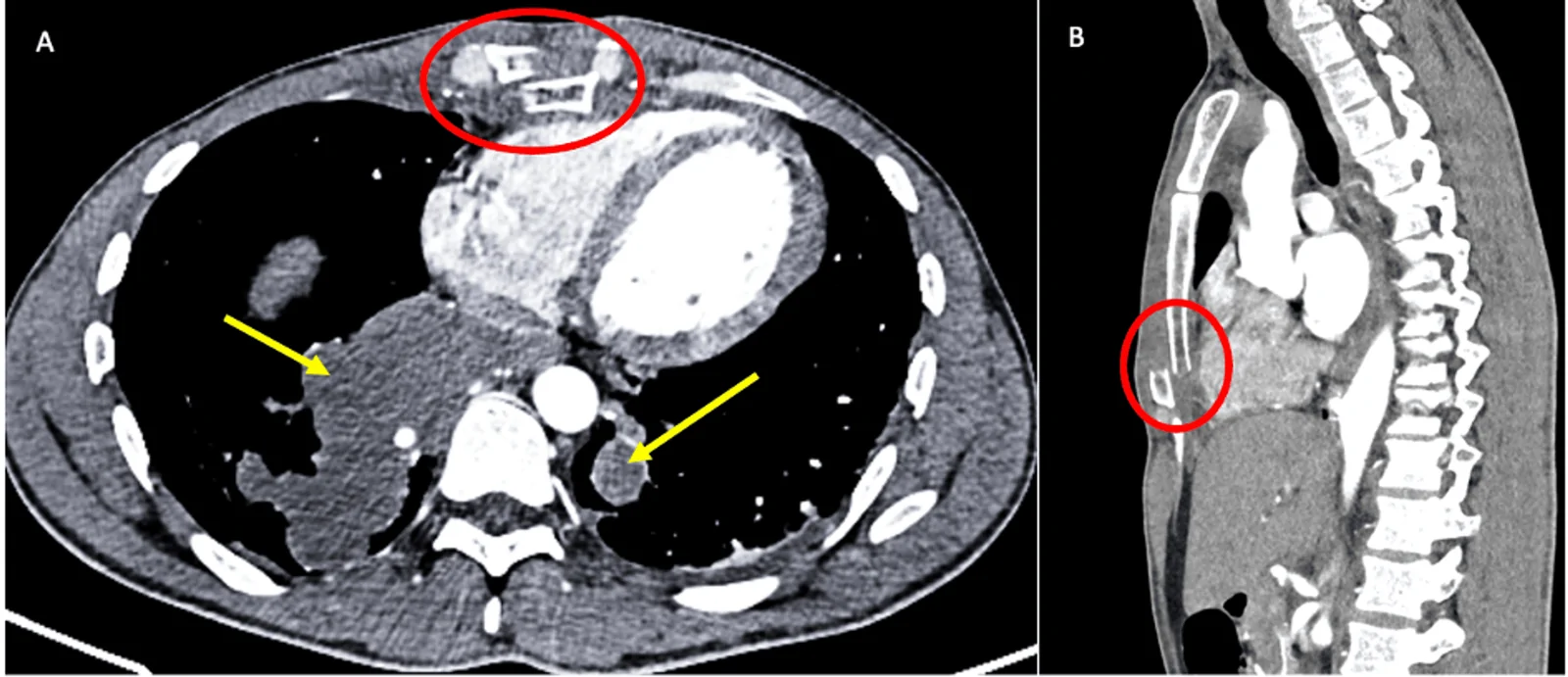
Axial (A) and sagittal (B) computed tomography (CT) angiography images showing the displaced sternal fracture (red circle) and heterogenous tissular and cystic bilateral lower lobe consolidation, larger in the right side (yellow arrows).

**Figure 2:**
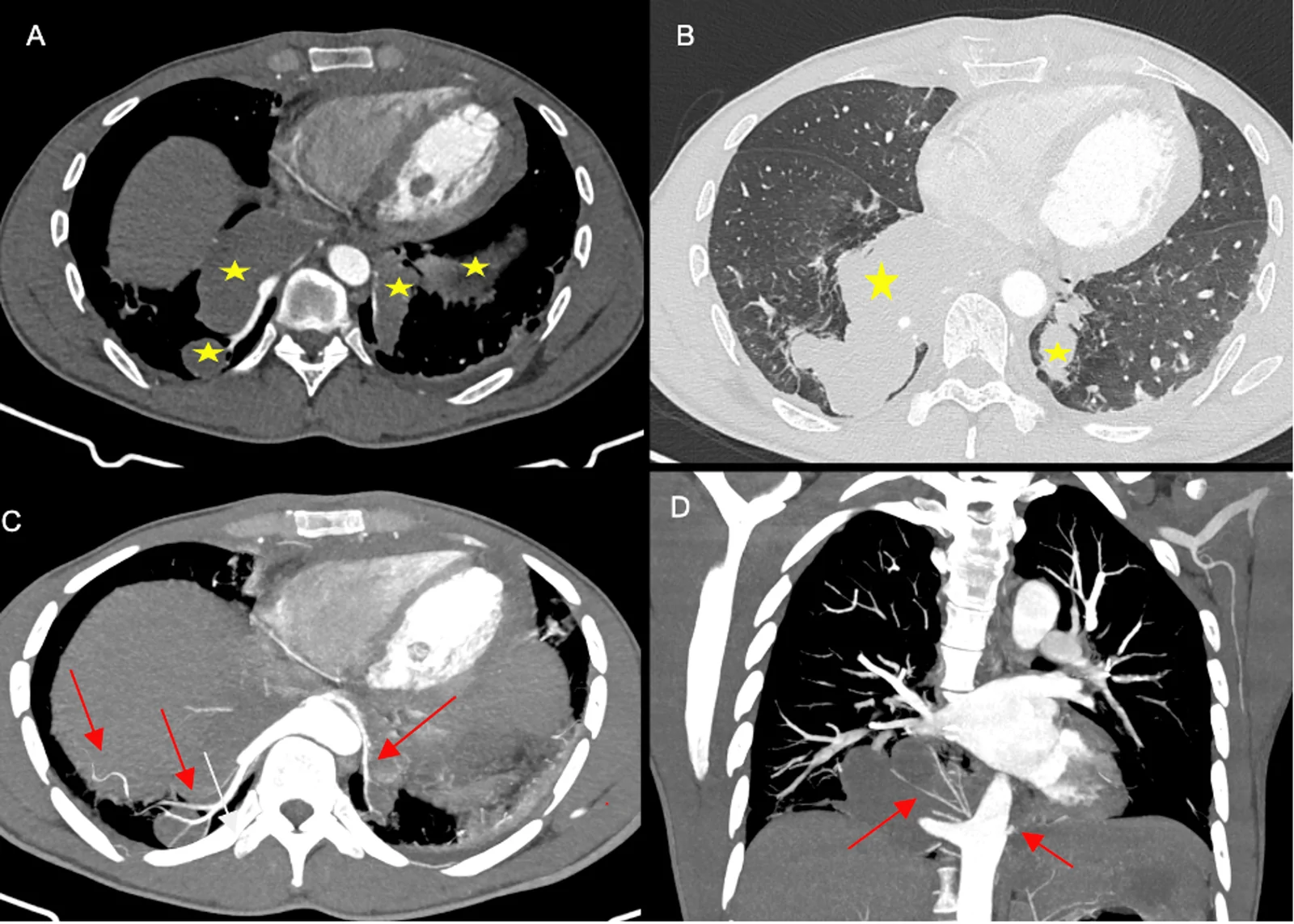
Axial CT angiography images obtained with soft-tissue (A) and lung (B) window settings show bilateral heterogenous tissular and cystic consolidative lesions in the basal segments of the lower lobes (yellow asterisks). Axial (C) and coronal (D) maximum-intensity-projection (MIP) images demonstrate aberrant systemic arterial branches (red arrows) originating from the thoracic aorta and supplying these lesions, confirming the diagnosis of bilateral intralobar pulmonary sequestration. CT: Computed tomography.

An unenhanced thoracic CT performed in the traumatic context revealed a displaced sternal fracture, together with a bilateral, heterogeneous tissular mass with cystic components in the lower lobes. Given the atypical pseudotumoral appearance of these lesions and their unusual bilateral distribution, a dedicated contrast-enhanced CT angiography of the chest was obtained. It demonstrated that both lesions were supplied by aberrant arterial branches arising from the descending thoracic aorta - a finding pathognomonic of pulmonary sequestration (Figures 1, 2). Multiplanar and maximum-intensity-projection (MIP) reconstructions allowed precise depiction of the origin and course of the aberrant arteries. The main diagnostic challenge was the pseudotumoral appearance of the lesions, which could readily have been mistaken for a neoplasm with necrotic components or a chronic infectious process; no laboratory or technical limitation hampered the work-up.

The final diagnosis was bilateral intralobar pulmonary sequestration, based on the intraparenchymal location of the lesions within the lower lobes, the absence of a separate pleural envelope, and the demonstration of an aberrant systemic arterial supply from the thoracic aorta. The differential diagnoses initially considered were a neoplastic lesion, a chronic or necrotizing infection, a pulmonary contusion, and other congenital lung malformations. The prognosis of pulmonary sequestration is generally excellent after definitive treatment; when left untreated, the main risks are recurrent infection, significant hemoptysis, and, rarely, pulmonary hypertension due to chronic systemic-to-pulmonary shunting.

The displaced sternal fracture was treated conservatively with analgesia and close respiratory monitoring, without surgical fixation. For the pulmonary sequestration, surgical resection (lobectomy or segmentectomy with prior ligation of the aberrant systemic artery) was proposed after multidisciplinary discussion as the standard treatment for this condition.

The post-traumatic course was uneventful, with progressive resolution of the chest pain under conservative management. The patient declined the proposed surgical resection of the sequestration. He was therefore enrolled in regular clinical and radiological follow-up in order to detect any potential complication, such as recurrent infection or hemoptysis. At the time of reporting, he remained stable, with no new respiratory symptom beyond his long-standing cough. After the potential benefits and complications of both surgery and surveillance had been explained to the patient, he chose radiological monitoring and stated that he understood the importance of regular follow-up.

## Discussion

Two features make this case particularly instructive. First, its bilaterality: pulmonary sequestration is itself an uncommon congenital lung malformation, and synchronous bilateral involvement is exceedingly rare, with only isolated cases reported in the literature [6,7]. Second, the lesion remained clinically unrecognized for 35 years and was discovered incidentally on CT performed for an unrelated sternal fracture. The patient’s longstanding, previously unexplored cough may retrospectively have represented its only clinical manifestation, possibly related to recurrent or low-grade pulmonary infections. These distinctive features frame the key issues addressed in this discussion: the embryologic basis of pulmonary sequestration, its capacity to remain clinically silent for decades, and the importance of its recognition despite its rarity.

Pulmonary sequestration owes its reputation as a diagnostic impostor to the fact that its parenchymal appearance is entirely non-specific. In adults, intralobar sequestration (ILS) - which represents 75-85% of cases - is located within a normal pulmonary lobe, sharing the visceral pleura of the adjacent lung, typically draining into pulmonary veins, and sometimes presents aberrant connections with airways. It shows a well-documented predilection for the left lower lobe [1,4,8]. It most often presents on CT as a soft-tissue mass or a cystic lesion filled with fluid, air, or both. These pseudotumoral appearances are readily mistaken for a neoplasm or a chronic necrotizing infection, and misdiagnosis or long diagnostic delay is the rule rather than the exception [4]. ELS, by contrast, has its own pleural envelope, no airway connection, and systemic venous drainage through the azygos or caval systems; it usually appears as a homogeneous, well-defined mass that, being insulated from the airway, almost never contains air, and it typically declares itself in the neonatal period or is associated with other congenital anomalies [2,8]. What rescues the diagnosis in every subtype is a single unifying clue that no tumor or pneumonia can imitate: an anomalous systemic artery, usually from the thoracic or abdominal aorta, feeding the lesion. This is precisely the sign that resolved our case, where aberrant branches from the descending thoracic aorta converted an alarming bilateral pseudotumoral picture into a confident, benign diagnosis.

The origin of that aberrant vessel remains a genuinely unsettled question, and it is more than an academic one. The classical view, tracing back to Pryce, holds that an accessory lung bud arising from the primitive foregut retains its embryonic systemic supply; whether it buds before or after the pleura forms then dictates a shared (ILS) or a separate (ELS) pleural covering [2,3,8]. Yet the frequency with which ILS is diagnosed only in adulthood, often against a background of recurrent infection, has led some authors to favor a mixed or partly acquired mechanism, in which chronic inflammation recruits a hypertrophied systemic vessel into a previously normal lung [4,8]. This debate frames sequestration as one end of a continuum of bronchopulmonary foregut malformations rather than a fixed entity, a spectrum that also encompasses hybrid lesions sharing features with congenital pulmonary airway malformation [5,8]. Against this backdrop, a bilateral case is conceptually striking, since it implies either two independent developmental accidents or a single insult acting on both lung buds - an observation that, however rare, keeps the acquired-versus-congenital question open.

The clinical stakes of the diagnosis flow directly from that systemic blood supply. Because the sequestered tissue is perfused at systemic rather than pulmonary arterial pressure, erosion of the feeding vessel can produce hemoptysis that is disproportionately severe, occasionally massive, and even complicated by hemothorax [9]. Chronic high-flow shunting can, more rarely, impose a volume load culminating in pulmonary hypertension [4,8]. This same feature carries a warning that our trauma context makes concrete: an unrecognized systemic feeder is a latent source of intrathoracic hemorrhage after chest injury and a well-known hazard in the operating room, where inadvertent division of the aberrant artery - which may retract into the mediastinum - can precipitate torrential bleeding. For this reason, secure identification and ligation of the vessel before any parenchymal resection are mandatory [8,10], and mapping it preoperatively is one of the most valuable services imaging can render.

It is here that CT angiography has become indispensable, having largely displaced conventional catheter angiography as the reference examination. Acquired in the arterial phase, it distinguishes the subtypes by simultaneously depicting lesion location, arterial supply, and venous drainage, while multiplanar, MIP, and volume-rendered reconstructions trace the exact origin, course, and caliber of the aberrant artery - the very roadmap the surgeon or interventional radiologist requires [5,10]. Prenatal ultrasound retains an important role antenatally, where it can already identify the feeding vessel, though its yield falls in neonates and adults; MRI and MR angiography offer a radiation-free alternative that likewise displays the systemic supply [8]. Once the diagnosis is secure, surgical resection remains the treatment of choice for symptomatic disease, increasingly complemented or replaced by endovascular embolization in high-risk patients or as a preoperative adjunct, albeit with a residual risk of incomplete occlusion and recurrence [8,10]. Whether an asymptomatic adult should nonetheless undergo resection is debated: many advocate intervention to forestall infection and hemoptysis, and our patient’s decision to decline surgery in favor of surveillance reflects the legitimate tension between a benign present and an unpredictable future - a choice that, in a bilateral case, also spares him bilateral parenchymal loss but demands vigilant follow-up.

The main strength of this report is the documentation of an exceptionally rare bilateral ILS and the demonstration that CT angiography, through its multiplanar and MIP reconstructions, can establish the diagnosis outright by revealing the aberrant systemic supply - here as a wholly incidental finding during trauma imaging. Its limitations should be stated plainly. Because the patient declined surgery, no histopathological confirmation was obtained, and the diagnosis rests on characteristic imaging alone. The venous drainage of each lesion was not fully characterized, so the intralobar classification is inferred from the intraparenchymal location and the absence of a separate pleura rather than from demonstrated pulmonary venous return. Finally, as a single observation with limited follow-up, this case cannot establish the long-term outcome of conservatively managed bilateral sequestration, which remains an open and worthwhile question.

## Conclusions

Pulmonary sequestration, although uncommon in adults, should be considered in the differential diagnosis of any atypical basal pulmonary lesion, particularly when the abnormality is bilateral. CT angiography is the cornerstone of the diagnosis, directly demonstrating the aberrant systemic arterial supply and providing the anatomical information required for treatment planning. Recognizing this entity is essential to avoid confusion with a tumor and to prevent unnecessary treatment or invasive procedures.
